# Efficacy of artificial intelligence-driven conversational agents (chatbots) for mental health promotion among university students: a systematic review and meta-analysis

**DOI:** 10.3389/fpsyt.2026.1824378

**Published:** 2026-10-09

**Authors:** Chen Shao, Xiaochen Chen, Shengnan Qiao, Ang Li, Shipeng Liu, Yanglong Chen

**Affiliations:** 1 School of Intelligent Engineering, Shandong Management University, Jinan, China; 2 Library, Shandong Management University, Jinan, China

**Keywords:** anxiety, artificial intelligence, chatbot, depression, digital phenotyping, mental health, university students

## Abstract

**Background:**

University students experience substantial mental-health burdens, while access to timely support remains uneven. AI conversational agents may provide low-threshold support, but the evidence base has expanded rapidly and prior reviews have often combined different age groups, intervention types, outcome domains, and comparator conditions.

**Objective:**

To evaluate the effects of AI-mediated conversational interventions on depressive and anxiety symptoms in university students, while distinguishing outcome domains, comparator intensity, intervention architecture, and the evidential requirements for standardized post-intervention effect estimation.

**Methods:**

We updated the search in PubMed, Embase, PsycINFO, CENTRAL, and Web of Science to the revised search date and rebuilt an auditable cross-database record ledger. The updated workflow included 3,786 exported records, 451 DOI/PMID duplicates, 41 confirmed title/author duplicates, and 3,294 unique records screened. All title/abstract records completed a two-reviewer screening and consensus chain. Forty-six update-search records entered research-level/full-text assessment. Randomized controlled trials were eligible for causal quantitative synthesis; relevant feasibility, acceptability, usability, and non-comparable controlled studies were retained narratively. Standard post-intervention analyses used Hedges g, REML random-effects models, and Hartung-Knapp adjustment, and were restricted to attributable two-group post-intervention data.

**Results:**

In the final locked non-active/low-intensity comparator analysis, three depression comparisons favored conversational interventions (k=3; Hedges g=-0.413, 95% CI -0.557 to -0.268; I2 = 0.0%) and four anxiety comparisons also favored them (k=4; Hedges g=-0.435, 95% CI -0.603 to -0.268; I2 = 2.1%). In the separate active conversational-control analysis, two MYLO comparisons did not show a reliable incremental effect for depression (k=2; g=0.165, 95% CI -0.580 to 0.911) or anxiety (k=2; g=0.190, 95% CI -0.556 to 0.937). The evidence base remained small, clinically heterogeneous, and limited by attrition, self-reported outcomes, and uncertainty regarding generalizability.

**Conclusions:**

AI conversational interventions may improve depressive and anxiety symptoms relative to non-active or low-intensity comparators in university students, but the certainty of evidence is low and estimates are based on few comparisons. Evidence against active conversational controls is very uncertain. Outcome-domain differences and architecture-related claims should be treated as hypothesis-generating. Secure, consent-based, human-supervised implementation and objective outcome assessment are priorities for future research.

**Systematic review registration:**

https://www.crd.york.ac.uk/PROSPERO/view/CRD420261323382, identifier CRD420261323382.

## 1 Introduction

### 1.1 Background: the mental health crisis among university students

Globally, the university student population is facing an increasingly severe mental health crisis. A multitude of compounding factors—including academic pressure, social adaptation challenges, and financial burdens—have contributed to the high prevalence of psychological issues such as depression and anxiety (1, 2). Traditional mental health service models struggle to meet the massive and urgent needs of this demographic due to inherent limitations such as restricted resources, poor accessibility, and pervasive social stigma (3, 4).

### 1.2 The role of AI conversational agents in digital psychiatry

Against this backdrop, digital mental health interventions, particularly artificial intelligence (AI) conversational agents (chatbots), have emerged as a promising solution (5). These technologies demonstrate substantial application potential owing to their anonymity, convenience, cost-effectiveness, and high scalability (6). By delivering personalized support grounded in evidence-based psychotherapeutic frameworks (e.g., Cognitive Behavioral Therapy) (Daley et al., 2020), AI conversational agents transcend temporal and spatial constraints, providing students with immediate and confidential mental health care (7).

### 1.3 Why this review remains needed in a rapidly evolving evidence base

The present review is not intended to replace earlier syntheses or to claim that previous reviews were unnecessary. Existing reviews address related but non-identical questions, and their differences in population, intervention definition, outcome selection, comparator, and study-design eligibility affect the interpretation of their pooled estimates (8).

First, the population boundaries differ. Chen et al. (9) and Feng et al (7) examined adolescents and young adults, while Feng et al (10) examined young people aged 12–25 years; these populations include, but are not limited to, university students. Li et al. (11) likewise synthesized chatbot interventions among young people, and Li et al. (12) included participants ranging from children and adolescents to adults and older adults. Leung et al. (13) focused on people in Asia across age groups rather than university students specifically. These broader age and geographic scopes are valuable for understanding the field, but they may combine developmental stages, educational contexts, help-seeking patterns, and campus-service environments that are not interchangeable with those of university students.

Second, the intervention boundaries differ. Zhang et al. (14) specifically synthesized generative or hybrid generative chatbots. Hang et al. (15) restricted the intervention to CBT-based, NLP-enabled conversational agents. These focused definitions provide useful evidence about particular technical or therapeutic classes, but they do not represent the full range of rule-based, generative, and hybrid participant-facing systems considered in the present review. By contrast, Lau et al. (16) evaluated AI-based psychotherapeutic interventions more broadly, Ferrari et al. (17) evaluated digital psychological interventions among university students, and Lattie et al. (18) reviewed digital mental-health interventions for depression, anxiety, and psychological well-being among college students. The latter categories may include digital interventions in which a conversational agent is not the active therapeutic component. Lai et al. (19) addressed AI in undergraduate health-professions education, which is relevant to the wider educational-AI landscape but does not answer a mental-health intervention question. Amaro et al. (20) described a protocol for a broad review of mental-health-promoting intervention models in university students; it is therefore not a completed chatbot-specific effectiveness synthesis.

Third, the outcome and comparator estimands are not identical. Several reviews combine depression, anxiety, stress, distress, wellbeing, positive or negative affect, and health behaviors in a single evidence landscape or across related analyses (5, 7, 10–12, 16, 21). Sohn et al. (22) focused specifically on depressive and anxiety symptoms and therefore represents a particularly close outcome-level comparison, but included a much broader population and 39 studies. He et al. (5) and Ferrari et al. (17) illustrate the importance of comparator and outcome selection by including different active, information, passive, or digital-intervention conditions. An effect relative to a waitlist or low-intensity information condition does not answer the same question as an incremental effect relative to another conversational agent. The present review therefore synthesizes depression and anxiety separately and stratifies non-active or low-intensity comparators from active conversational controls.

The closest population-and-intervention comparison is Nyakhar and Wang (23), which focused on AI chatbots among college students. This review makes an important timely contribution, but it was explicitly designed as a rapid systematic review, searched four databases, included nine studies, and used single-reviewer title/abstract screening with dual verification. The present review extends that evidence base through an updated search to 24 August 2026, a two-reviewer screening and consensus workflow, explicit report-to-study linkage, and separation of qualitative eligibility from quantitative meta-analytic eligibility. It also distinguishes depression from anxiety, separates immediate post-intervention from follow-up outcomes, and audits whether each quantitative estimate is attributable to the conversational intervention and based on compatible two-group post-intervention data.

The closest outcome-level comparison is Sohn et al. (22), which synthesized chatbot effects on depression and anxiety using 39 studies. Its broader scope is important, but the inclusion of diverse populations and control conditions means that its pooled estimates answer a general chatbot-effectiveness question rather than the university-student-specific question addressed here. Chen et al. (9) provides another close comparator because it restricted its synthesis to depression and anxiety and randomized trials, but its adolescent-and-young-adult population and earlier search date differ from the present review. Feng et al (10) similarly studied 12–25-year-olds and multiple mental-health outcomes, whereas the present review isolates university or college students and applies a stricter attribution rule for standard post-intervention SMD pooling. Zhang et al. (14) is closest in its explicit effect-size eligibility and generative/hybrid focus, but it does not cover the full architecture range or the university-student population of the present review.

Accordingly, the incremental contribution of this review is deliberately limited and specific: a current, university-student-focused, outcome-specific, comparator-stratified, and data-source-audited synthesis of participant-facing AI-mediated conversational interventions. The review does not claim that its narrower scope makes broader reviews less useful. Rather, it makes population, intervention architecture, comparator intensity, timepoint, report linkage, and effect-size provenance visible so that estimates from non-equivalent questions are not combined as though they represented the same intervention effect. A detailed comparison of these prior reviews with the present review is provided in **Supplementary Table S1**.

## 2 Methods

### 2.1 Study design and registration

This systematic review and meta-analysis was conducted in strict adherence to the Preferred Reporting Items for Systematic Reviews and Meta-Analyses (PRISMA) guidelines (24). The pre-specified study protocol was prospectively registered on the International Prospective Register of Systematic Reviews (PROSPERO) under the registration number CRD420261323382 (25).

### 2.2 Search strategy

We searched PubMed, Embase, PsycINFO, CENTRAL, and Web of Science from each database’s inception, where supported by the database, to the updated search date documented in the supplementary search log. Search terms combined population terms (university students, college students, undergraduates, and related terms), intervention terms (artificial intelligence, chatbot, conversational agent, virtual coach, generative AI, and related terms), and mental-health outcome terms (depression, anxiety, psychological distress, mental health, and related terms). Reference lists of relevant systematic reviews and eligible full-text reports were also checked.

The updated search was undertaken because the manuscript had originally been completed and submitted approximately six months earlier, during a period in which AI conversational-agent research expanded substantially. The update was therefore a complete search and screening exercise rather than a limited citation refresh.

The primary quantitative evidence base was restricted to randomized controlled trials to reduce confounding in estimates of comparative efficacy. Relevant quasi-experimental, single-arm, feasibility, acceptability, usability, and mixed-methods studies were not silently discarded: they were retained for narrative or scope-limited synthesis when they addressed the target population and intervention. They were not pooled as randomized controlled effects.

No language restriction was applied at the database retrieval stage. Any language-related limitation during full-text assessment is reported in the supplementary screening log. Grey literature and trial registries were considered as sources for identifying potentially relevant studies and ongoing or completed records; records without outcome results were logged separately and were not treated as completed evidence. The search strategy was checked against known eligible reports and the reference lists of relevant reviews. Nevertheless, database coverage, indexing differences, language availability, and unpublished-study retrieval may leave residual publication and language bias.

### 2.3 Eligibility criteria and intervention definition

Eligible interventions were participant-facing AI-mediated conversational systems intended to provide mental-health support, prevention, or treatment. Systems could be rule-based, generative, or hybrid. Architecture was extracted as a descriptive characteristic: rule-based systems used predefined scripts or decision rules; generative systems generated responses through a large language model or comparable generative engine; and hybrid systems combined scripted therapeutic modules with generative or natural-language components. Hybrid systems were not forced into either the rule-based or generative category.

The functional intervention definition was used because therapeutic purpose and participant-facing conversational delivery were central to the review question, whereas architecture is an evolving technical property. Architecture type, therapeutic framework, duration, contact frequency, human support, and comparator were extracted separately. Because the number of comparable studies was small and intervention components were strongly confounded with year, country, framework, and comparator, architecture-specific subgroup meta-analysis was considered exploratory and was not used to make causal claims.

### 2.4 Data extraction and quality assessment

Two reviewers independently assessed records and resolved discrepancies by consensus, following the review methods prespecified in the PROSPERO record and reported in accordance with PRISMA 2020 (24). Reports were linked at the study level using DOI, trial registration, author/sample characteristics, intervention identity, comparator structure, and other report-level information. Multiple reports from the same trial were not counted as independent studies. The primary Shoshani JAMA Network Open report and its associated KAI report were treated as reports from one research study (26). Bird and Gaffney were treated as two independent MYLO student trials (27, 28).

Extracted variables included participant characteristics, country, recruitment context, intervention architecture, therapeutic framework, intervention duration, contact schedule, human involvement, comparator, outcome instrument, post-intervention and follow-up timepoints, sample size, mean, standard deviation, adjusted estimates, attrition, and analysis population.

Risk of bias was assessed with RoB 2 across randomization, deviations from intended interventions, missing outcome data, outcome measurement, and selection of the reported result (29). Lack of participant blinding was considered in the interpretation of deviations and self-reported outcome measurement. Certainty of evidence was assessed using GRADE, taking risk of bias, inconsistency, indirectness, imprecision, and publication bias into account (30).

### 2.5 Statistical analysis

Depression and anxiety were synthesized separately. Hedges g was selected as the standardized mean difference because it applies a small-sample correction that is relevant to the small student trials included in this review (31). Negative values indicate lower symptoms in the conversational-agent group.

The standard post-intervention SMD analysis required an attributable conversational-agent intervention, an eligible comparator, and compatible two-group post-intervention mean, standard deviation, and sample-size data. Adjusted means accompanied by standard errors were not treated as raw standard deviations. Within-group change effects were not converted into between-group post-intervention SMDs. Studies with incompatible reporting were retained in the qualitative synthesis or presented as scope-limited evidence.

REML random-effects models with Hartung-Knapp adjustment were used because clinical and methodological heterogeneity was expected and the number of comparable studies was small (31, 32). Non-active or low-intensity comparators were analyzed separately from active conversational comparators. Immediate post-intervention and follow-up outcomes were not pooled. Leave-one-out analyses and risk/attrition-focused sensitivity analyses were conducted where the available data supported them. Architecture, duration, and therapeutic framework were summarized descriptively; the small number of studies did not support reliable meta-regression.

Funnel plots and formal small-study tests were not used when fewer than 10 comparisons contributed to an outcome, because such tests have limited interpretability in small meta-analyses.

## 3 Results

### 3.1 Literature search and study selection

The updated search produced 3,786 exported records. After removal of 451 DOI/PMID duplicates and 41 confirmed title/author duplicates, 3,294 unique records entered title/abstract screening. All 3,294 records completed the two-reviewer screening and consensus chain. A total of 3,248 records were excluded at title/abstract level. Forty-six update-search records entered research-level/full-text assessment.

Among these 46 records, 24 had verified full text, 12 were associated reports mapped to existing verified studies, 6 were excluded at the research-level stage because the abstract or bibliographic record was sufficient to establish ineligibility, and 4 were registry records or records without outcome results. These categories are mutually exclusive in the update-search log. Historical representative reports were added to the working study registry to ensure coverage of previously locked studies, but those five backfilled reports are not substituted for the 46 update-search records in the PRISMA update-search node (**Figure 1**) (24).

**Figure 1:**
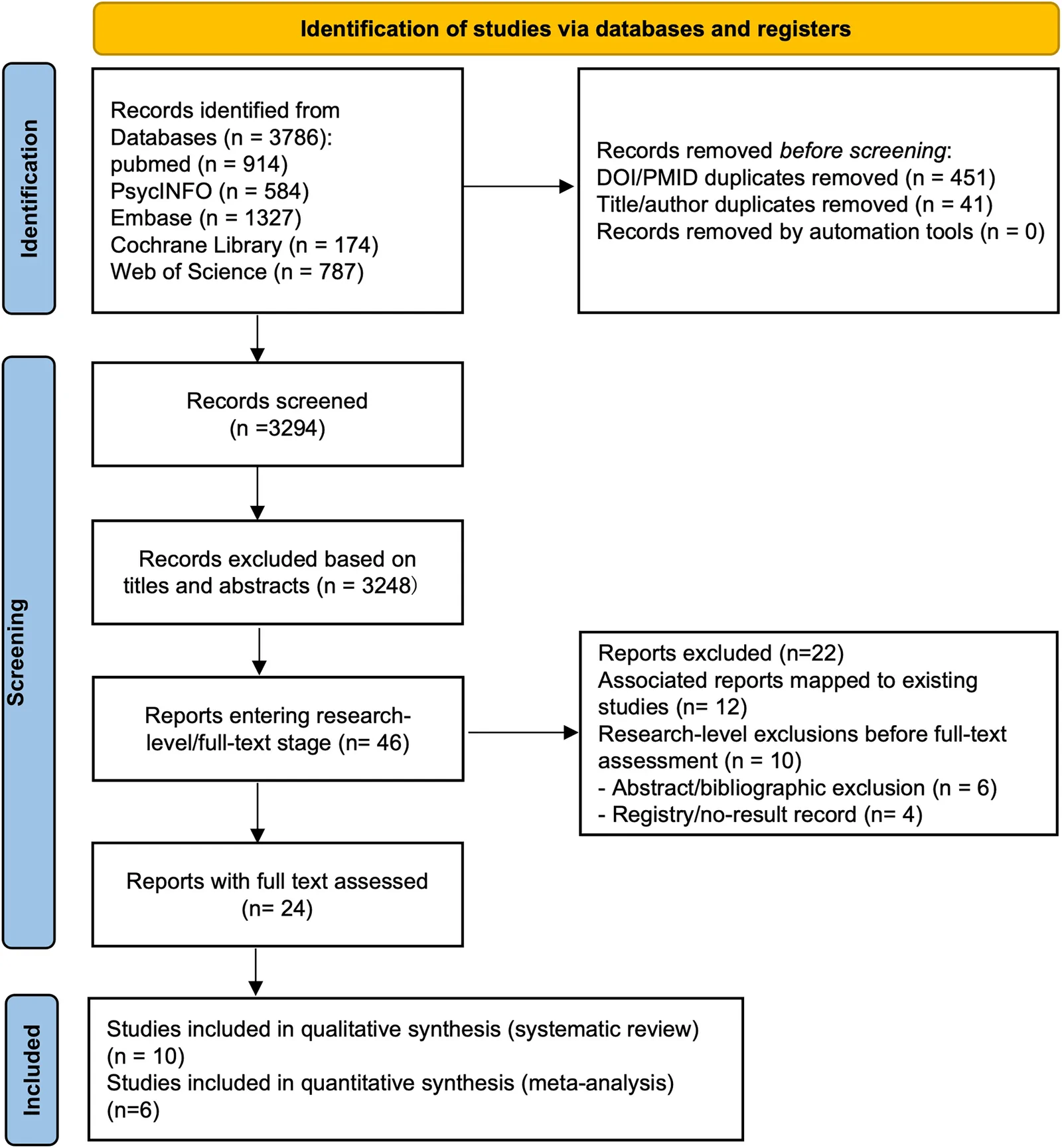
PRISMA flow chart.

### 3.2 Characteristics of included studies

The current working independent-study registry contains 23 study clusters, including randomized studies of Woebot, Tess, XiaoNan, MISHA, UP chatbot, MYLO, KAI, Psy-Bot, and other feasibility or scope-limited conversational interventions (26–28, 33–39). Twenty-two clusters have a qualitative, effectiveness, feasibility, acceptability, or scope-limited synthesis disposition; one associated technical report does not constitute an independent clinical study. The evidence spans rule-based, generative, and hybrid systems and CBT, mindfulness, psychoeducation, problem-solving, and mixed therapeutic frameworks. Intervention periods range from approximately 7 days to 16 weeks, and comparators include waitlists, informational or psychoeducational materials, low-intensity digital comparators, and active conversational agents.

The diversity of interventions and contexts is clinically important but limits direct comparability. The study registry therefore reports architecture, therapeutic framework, duration, comparator, and outcome separately rather than treating them as interchangeable features of one intervention class (**Table 1**).

**Table 1:** Characteristics of primary randomized controlled trials of AI-mediated conversational interventions among university or college students.

Study	Country and population	Design	Conversational intervention/architecture	Comparator	Mental-health outcomes	Assessment timepoint(s)	Role in this review
27	United Kingdom; university students and staff/healthy volunteers	RCT	MYLO; rule-based conversational problem-solving agent	ELIZA active conversational agent	DASS-21 depression and anxiety	Post-intervention; 2-week follow-up	Active conversational comparator meta-analysis
28	United Kingdom; University of Manchester students	Pilot RCT	MYLO; rule-based conversational problem-solving agent	ELIZA active conversational agent	DASS depression and anxiety	Post-intervention; follow-up	Active conversational comparator meta-analysis
33	United States; college-community young adults (18–28 years)	RCT	Woebot; rule-based/NLP CBT conversational agent	NIMH psychoeducational information ebook	PHQ-9 depression; GAD-7 anxiety	2–3 weeks	Narrative synthesis; effect reporting not compatible with standard post SMD
39	China; college students (17–21 years)	Three-arm RCT	XiaoE; RASA/CBT chatbot	E-book and general chatbot	PHQ-9 depression	1 week; 1-month follow-up	Narrative synthesis; adjusted mean/SE not used as raw SD
34	Argentina; university students	Pilot RCT	Tess; scripted/rule-based chatbot	Psychoeducation ebook	PHQ-9 depression; GAD-7 anxiety	8 weeks	Narrative synthesis; anxiety sensitivity evidence only because of high attrition
35	China; university students (N = 83)	RCT	XiaoNan; RASA/CBT chatbot	Bibliotherapy	PHQ-9 depression; GAD-7 anxiety	16 weeks	Narrative synthesis; reported adjusted effect only
36	Switzerland; university students with stress	Pilot RCT	MISHA; rule-based stress-management coaching agent	Waitlist	PHQ-9 depression; GAD-7 anxiety	Post-intervention (4–7 weeks)	Non-active/low-intensity comparator meta-analysis
38	China; university students	RCT	Psy-Bot; hybrid RASA/NLP CBT chatbot	Waitlist	CES-D-10 depression; GAD-7 anxiety	7 days	Narrative synthesis; depression reported change effect; no standard post SMD
37	Japan; university students	Four-arm RCT	Unified Protocol chatbot; generative/rule-guided hybrid	Chat-only and waitlist; prespecified AI vs waitlist comparison	ODSIS depression; OASIS anxiety	8 weeks; 12-week follow-up	Non-active/low-intensity comparator meta-analysis
26	Israel; university students with psychological distress (N = 995)	Three-arm RCT	KAI; generative LLM conversational agent	Waitlist and face-to-face group therapy	PHQ-9 depression; GAD-7 anxiety	12 weeks; 3-month follow-up	Non-active/low-intensity comparator meta-analysis (AI vs waitlist); group-therapy comparison narrative

### 3.3 Risk of bias assessment

Risk-of-bias judgments are presented in the RoB 2 table and traffic-light figure (**Figure 2**). The most recurrent concerns relate to inability to blind participants, self-reported outcomes, attrition, and incomplete or completer-based analyses. Klos et al. contributes anxiety data using week-8 completers and is considered high risk in the missing-outcome-data domain (34). These limitations reduce confidence in the magnitude of pooled effects.

**Figure 2:**
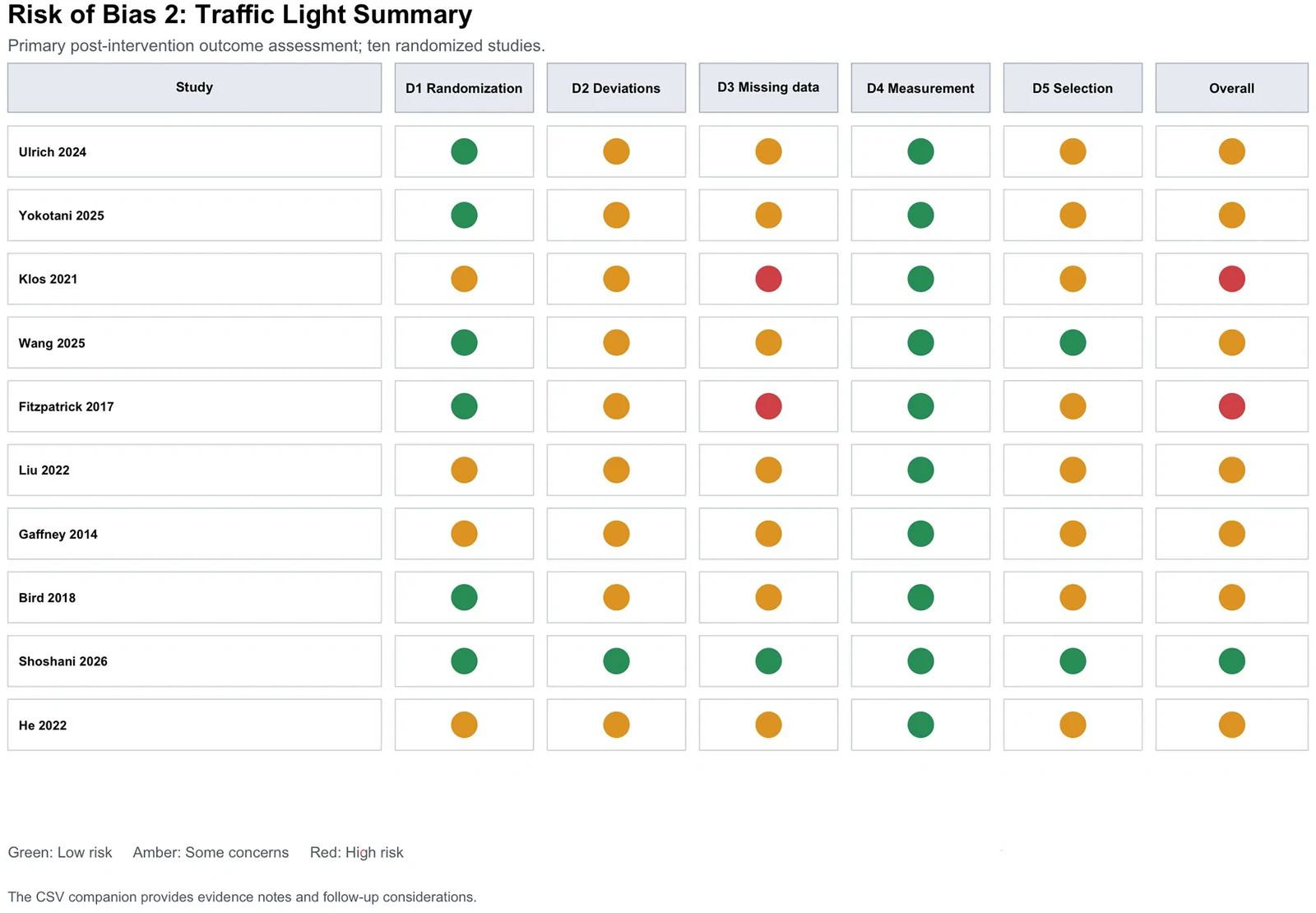
RoB 2 traffic-light summary for primary post-intervention outcomes.

GRADE certainty was rated as low for the non-active or low-intensity comparator evidence and very low for active conversational-comparator evidence. Random allocation does not eliminate concerns arising from missing data, subjective outcome measurement, small sample sizes, indirectness, or the limited number of independent comparisons. The GRADE certainty-of-evidence assessment is summarized in **Figure 3**.

**Figure 3:**
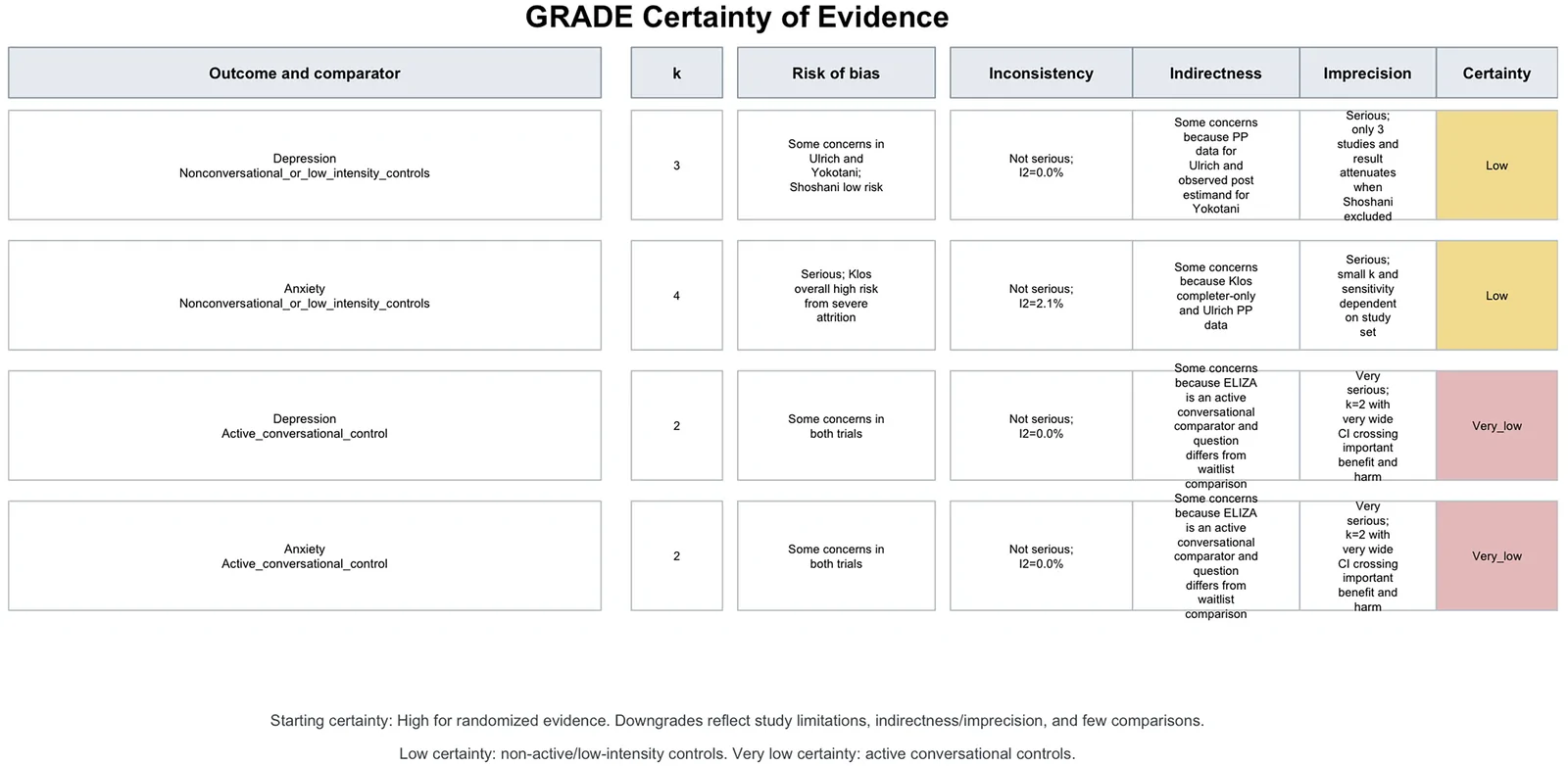
GRADE certainty-of-evidence summary.

### 3.4 Effect of AI interventions on depressive symptoms

In the final locked non-active/low-intensity comparator analysis, three depression comparisons contributed compatible post-intervention data (26, 36, 37). Conversational-agent interventions favored lower depressive symptoms (k=3; Hedges g=-0.413, 95% CI -0.557 to -0.268; I2 = 0.0%). This estimate should be interpreted as a short-term average across a small number of heterogeneous interventions and not as evidence that all chatbot architectures or therapeutic frameworks have equivalent effects (**Figure 4**).

**Figure 4:**
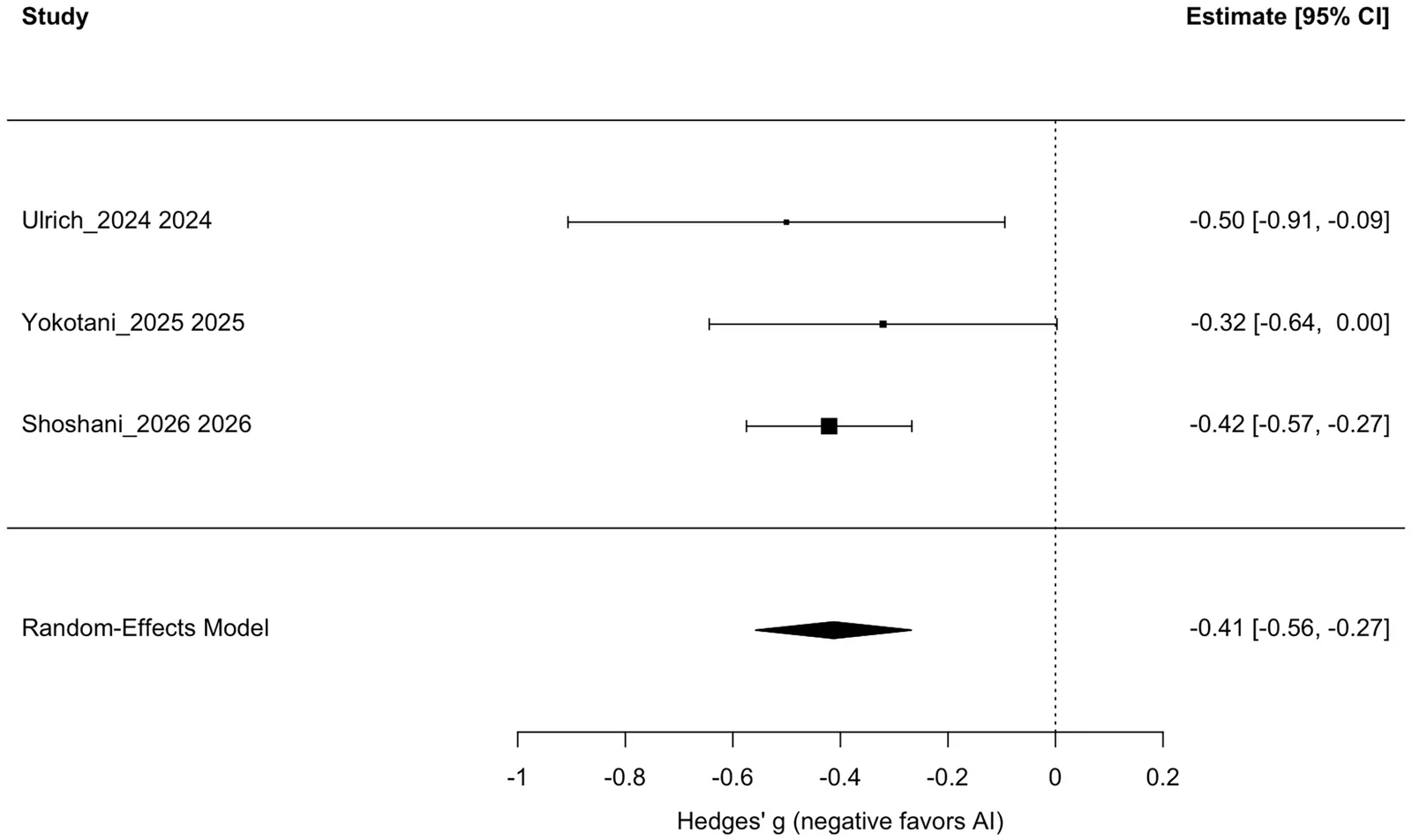
Forest plot of depressive symptoms for conversational interventions versus non-active or low-intensity controls.

### 3.5 Effect of AI interventions on anxiety symptoms

Four anxiety comparisons contributed compatible post-intervention data in the final locked non-active/low-intensity comparator analysis (26, 34, 36, 37). The pooled estimate favored conversational-agent interventions (k=4; Hedges g=-0.435, 95% CI -0.603 to -0.268; I2 = 2.1%). The small number of comparisons and the presence of attrition and self-report limitations mean that the estimate remains uncertain. It should not be interpreted as evidence that conversational agents are sufficient for acute anxiety or crisis care.

In the separate active conversational-control analysis, two MYLO comparisons did not show a reliable incremental effect for depression (k=2; Hedges g=0.165, 95% CI -0.580 to 0.911; 27, 28) or anxiety (k=2; Hedges g=0.190, 95% CI -0.556 to 0.937). These results emphasize that comparator intensity materially changes the interpretation of apparent efficacy (**Figure 5**).

**Figure 5:**
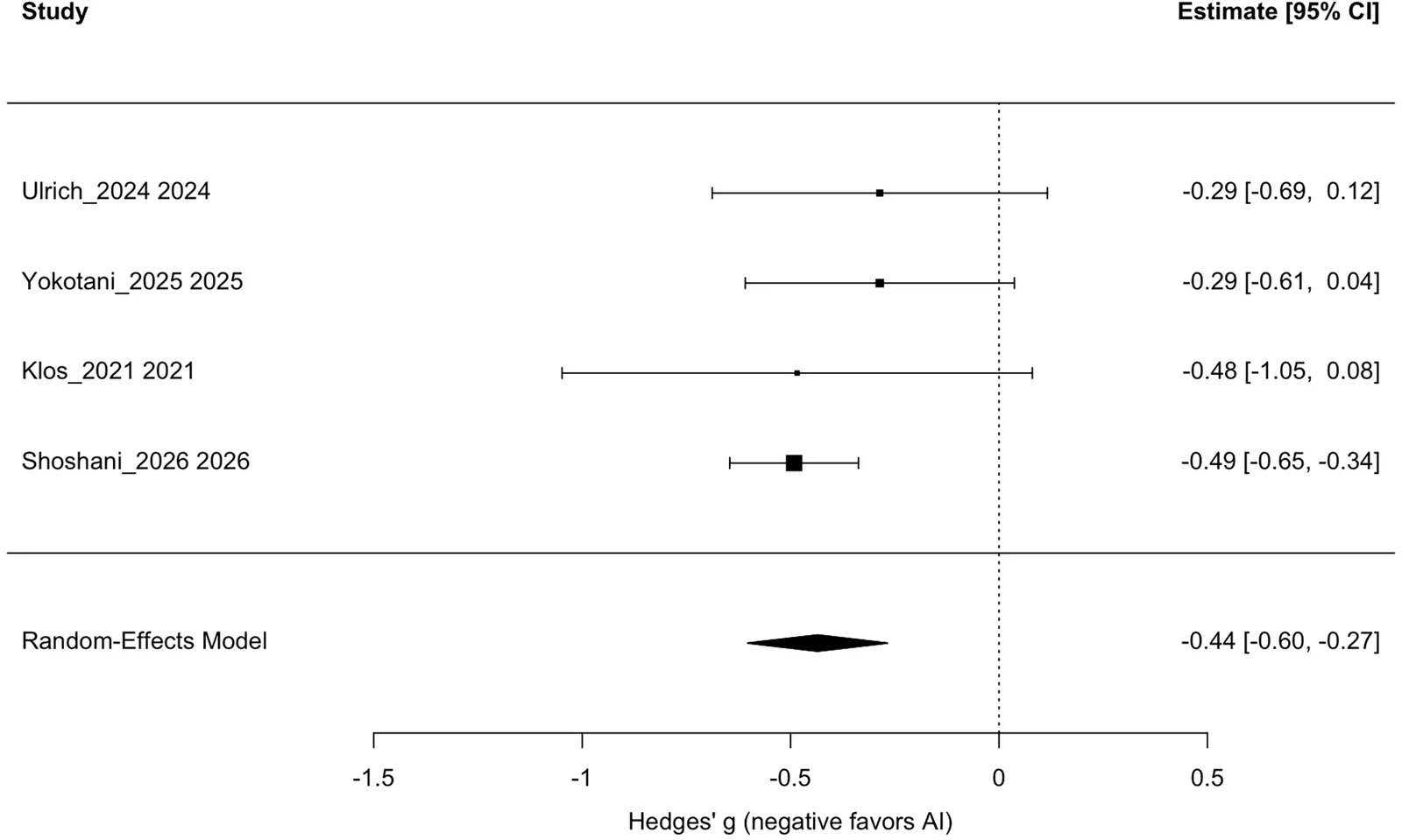
Forest plot of anxiety symptoms for conversational interventions versus non-active or low-intensity controls.

## 4 Discussion

### 4.1 Principal findings and relation to previous reviews

The systematic review provides a focused synthesis of AI-mediated conversational interventions for university students. Relative to non-active or low-intensity comparators, the final locked estimates favor improvement in both depressive and anxiety symptoms. However, the evidence base is small, and the available active conversational comparisons did not demonstrate a reliable incremental benefit. This pattern is broadly compatible with the direction of findings reported in several previous reviews, but the estimates should not be assumed to be numerically interchangeable because those reviews differ in population, intervention scope, outcomes, comparators, and search dates (5, 7, 9–12, 16, 22).

The closest population-and-intervention review, Nyakhar and Wang (23), focused specifically on college students and AI chatbots and included nine studies. Its rapid-review design provided a useful and timely synthesis, but its four-database search and single-reviewer initial screening differ from the two-reviewer, study-level, and auditable workflow used in the present review. The present review differs from it in several respects, including the updated search to 24 August 2026, explicit report-to-study linkage, separation of qualitative from quantitative eligibility, comparator stratification, separate depression and anxiety analyses, separation of post-intervention from follow-up outcomes, and auditing of compatible two-group post-intervention effect-size inputs. The closest outcome-level synthesis, Sohn et al. (22), included 39 studies of depressive and anxiety symptoms, but its broader population and comparator conditions address a general chatbot-effectiveness question. Chen et al. (9) and Feng et al. (10) are also close in their focus on younger populations, but neither is restricted to university students; Feng et al. (10) further included health behaviors and a broader set of mental-health outcomes. Zhang et al. (14) provided a complementary generative-AI-focused synthesis, while Hang et al. (15) provided a CBT/NLP-specific synthesis. Their conclusions cannot be directly generalized to all architectures included in the present review.

The principal added value of this review is therefore methodological and population-specific. It separates symptom domains, comparator intensity, timepoint, architecture, and effect-size provenance. It also makes explicit which reports are independent studies and which are associated publications. These distinctions matter because broader reviews may combine adolescents with university students, general digital interventions with chatbot interventions, or active and passive comparators in analyses that target different estimands (10, 13, 17, 19). The present synthesis does not eliminate the limitations of the underlying trials, but it makes the boundaries of each estimate more transparent.

### 4.2 Depression, anxiety, and clinical interpretation

The observed estimates for depression and anxiety should not be interpreted as definitive evidence of different therapeutic mechanisms. Depression and anxiety are related but distinct symptom domains, and differences in recruitment, baseline severity, measurement instruments, treatment framework, exposure, and attrition may contribute to apparent domain differences. The small number of studies prevents a reliable test of whether architecture or symptom domain modifies treatment response.

CBT-oriented conversational delivery may support structured reflection, psychoeducation, and behavioral activation, while acute anxiety may require rapid assessment, crisis-sensitive responses, and forms of support that cannot be provided safely by an unsupervised automated agent (33, 40). These are plausible clinical considerations, not causal explanations established by this meta-analysis.

### 4.3 Comparator, engagement, and expectancy effects

Effects relative to waitlists or low-intensity materials may include nonspecific attention, hope, novelty, expectancy, and increased monitoring, in addition to therapeutic mechanisms. Active conversational-control results are therefore particularly informative, although currently sparse and imprecise. User engagement may influence exposure and adherence, but engagement should not be assumed to mediate symptom change without prospective mediation analyses. Future trials should predefine engagement measures and distinguish therapeutic content, attention, social presence, and technical usability.

### 4.4 Heterogeneity, attrition, and generalizability

Intervention duration varied from approximately 7 days to 16 weeks, and therapeutic frameworks and architectures also differed. These differences limit the assumption that the pooled estimate represents a single standardized dose. The REML random-effects model and Hartung-Knapp adjustment account statistically for uncertainty but cannot remove clinical heterogeneity. With few studies, subgroup analyses and meta-regression are unstable; architecture and duration findings should therefore remain descriptive.

Attrition is a major limitation. Completer analyses may overrepresent participants who tolerated or benefited from the intervention. High attrition, differential dropout, and reliance on self-reported measures reduce certainty. The evidence also spans multiple countries and educational settings, but geographic diversity alone does not establish cultural generalizability. Language, stigma, digital access, campus services, socioeconomic conditions, and expectations about AI-mediated support may influence both engagement and outcomes.

### 4.5 Responsible scalability, digital phenotyping, and future research

Conversational agents may offer a scalable delivery channel, but scalability is not equivalent to proven population-level effectiveness. Implementation at university scale requires local language and cultural adaptation, accessibility, integration with existing services, clear human escalation pathways, quality monitoring, and safeguards against inequitable access.

We have deliberately reframed digital phenotyping as a future research possibility rather than a demonstrated clinical function. Interaction logs may contain potentially informative behavioral signals, but their validity, reliability, clinical utility, and fairness require prospective validation (40–42). Any secondary use of conversational data should require meaningful consent, data minimization, purpose limitation, secure storage and transmission, role-based access controls, transparent retention policies, human oversight, and a clear response plan for safety signals. Passive monitoring must never silently replace clinical assessment or informed participation.

Future randomized trials should preregister architecture, therapeutic framework, duration, engagement, safety, and missing-data plans, and should report AI interventions transparently using relevant reporting guidance (43). In addition to validated self-report scales, investigators should consider clinician-rated outcomes, behavioral indicators, physiological measures where ethically and technically justified, and longer-term follow-up. Active conversational comparators and head-to-head architecture studies are particularly important for separating therapeutic effects from attention, expectancy, and novelty.

## 5 Conclusions

Among university students, AI-mediated conversational interventions may reduce depressive and anxiety symptoms relative to non-active or low-intensity comparators, but the certainty of evidence is low and the number of independent comparisons is limited. Evidence from active conversational comparisons is very uncertain and does not establish a reliable incremental benefit. The results should not be generalized to crisis intervention, unsupervised clinical care, or all chatbot architectures.

The field now requires transparent, preregistered, adequately powered trials with active comparators, complete follow-up, robust missing-data handling, culturally responsive design, and objective or clinician-rated outcomes alongside self-report. Conversational data may support future research on digital phenotyping only under explicit consent, strict privacy and security protections, and human clinical oversight. 

## Data Availability

The original contributions presented in the study are included in the article/**Supplementary Material**. Further inquiries can be directed to the corresponding author.
